# Measuring energy expenditure in the intensive care unit: a comparison of indirect calorimetry by E-sCOVX and Quark RMR with Deltatrac II in mechanically ventilated critically ill patients

**DOI:** 10.1186/s13054-016-1232-6

**Published:** 2016-03-05

**Authors:** Martin Sundström Rehal, Erik Fiskaare, Inga Tjäder, Åke Norberg, Olav Rooyackers, Jan Wernerman

**Affiliations:** Department of Anesthesiology and Intensive Care Medicine, K32, Karolinska University Hospital Huddinge, Hälsovägen 13, 14186 Stockholm, Sweden; Division of Anesthesia and Intensive Care, Department of Clinical Sciences, Intervention and Technology (CLINTEC), Karolinska Institutet, Hälsovägen 13, 14186 Stockholm, Sweden

**Keywords:** Indirect calorimetry, Mechanical ventilation, Intensive care, Validation

## Abstract

**Background:**

Indirect calorimetry allows the determination of energy expenditure in critically ill patients by measuring oxygen consumption (VO_2_) and carbon dioxide production (VCO_2_). Recent studies have demonstrated variable performance of “breath-by-breath” instruments compared to mixing chamber technology. The aim of this study was to validate two modern devices (E-sCOVX and Quark RMR) against a reference method (Deltatrac II).

**Method:**

Measurements of VO_2_/VCO_2_ with the test and reference devices were performed simultaneously over a 20-min period in mechanically ventilated adult intensive care unit patients. Accuracy and precision of instruments were analyzed using Bland-Altman plots.

**Results:**

Forty-eight measurements in 22 patients were included for analysis. Both E-sCOVX and Quark RMR overestimated VO_2_ and VCO_2_ compared to Deltatrac II, corresponding to a 10 % higher mean resting energy expenditure. Limits of agreement of resting energy expenditure within ±2 standard deviations were ±461 kcal/24 h (±21 % expressed as percentage error) for ΔE-sCOVX–Deltatrac II and ±465 kcal/24 h (±22 %) for ΔQuark RMR–Deltatrac II.

**Conclusion:**

Both test devices overestimate VO_2_ and VCO_2_ compared to Deltatrac II. The observed limits of agreement are comparable to those commonly accepted in evaluations of circulatory monitoring, and significantly less than results from predictive equations. We hypothesize that the discrepancy between methods is due to patient/ventilator-related factors that affect the synchronization of gas and spirometry waveforms.

**Trial registration:**

Australian New Zealand Clinical Trials Registry, Trial ID ACTRN12615000205538. Date registered 3 March 2015.

## Background

Deciding the optimal provision of energy for patients in the intensive care unit (ICU) presents an ongoing challenge to clinicians. Several guidelines recommend a calorie delivery targeted to energy expenditure (EE) [1, 2]. Results from recent large randomized controlled trials indicate that lower energy targets may be acceptable during the first weeks of ICU stay, but there is still uncertainty regarding optimal targets for patients with pre-existing malnutrition or a prolonged course of critical illness [3–6]. Regardless of feeding strategy, setting individual caloric goals requires an estimation of EE. For this purpose clinicians have two principal tools. They can either use equations with inputs from various patient characteristics, or indirectly measure the metabolic rate from oxygen consumption (VO_2_) and carbon dioxide production (VCO_2_). Studies comparing predictive equations to indirect calorimetry (IC) in critically ill patients show a poor agreement between calculated and measured EE [7, 8]. Indirect calorimetry allows for accurate determination of EE, but widespread adoption of the technique has been limited due to the technical demands of measurements [9].

The Deltatrac Metabolic Monitor (Datex, Helsinki, Finland) was the first calorimeter validated in ICU patients, demonstrating high precision and accuracy [10]. It has since been considered the gold standard for gas exchange measurements during mechanical ventilation. Deltatrac is no longer in production, highlighting the need for alternatives. Several devices have recently been released on the market to meet this demand. A common feature in modern instruments is that gas exchange is measured “breath-by-breath”, a technology that requires software algorithms to synchronize spirometry and gas concentration measurements. This poses a particular challenge in the modern ICU, where deep sedation is rare and the respiratory pattern of patients can be highly variable. We have previously demonstrated varying limits of agreement and systematic bias between Deltatrac and two breath-by-breath instruments when applied to critically ill mechanically ventilated patients. These findings have been corroborated by other research groups [11–13]. This raises concerns about the reliability of modern calorimeters in this clinical setting. It is therefore crucial that new technology is evaluated in the context of its intended use before it is applied to guide patient care.

The aim of this study is to determine the level of agreement in gas exchange measurements between the E-sCOVX (GE, Helsinki, Finland), Quark RMR (Cosmed, Rome, Italy) and the Deltatrac II in mechanically ventilated ICU patients. We hypothesized that all three instruments would measure VO_2_, VCO_2_ and EE with equal precision and accuracy.

## Methods

This study was conducted in a twelve-bed mixed surgical/medical ICU of a tertiary referral hospital. It was approved by the regional ethical review board in Stockholm (2014/1778-31) and registered at Australia New Zealand Clinical Trials Registry (Trial ID ACTRN12615000205538). Patients and relatives were informed about the study orally and in writing before written informed consent was obtained. All mechanically ventilated patients ≥18 years of age were considered for recruitment. Exclusion criteria were: 1) gas leaks (chest tubes, pneumothorax, bronchoesophageal fistulas, etc; leaks registered by the ventilator <10 % of minute volume (MV) were accepted); 2) NO therapy or ECMO 3) fraction of inspired oxygen (FiO_2_) >0.60; 4) respiratory rate (RR) >35; and 5) absence of informed consent. Although continuous renal replacement therapy (CRRT)-induced CO_2_ diversion can interfere with the accurate determination of metabolic CO_2_ production, the effect on pulmonary gas exchange measurements should be identical for all instruments. Patients with CRRT were therefore eligible for inclusion providing flow rates and filtration remained constant during the study period. All patients were mechanically ventilated by Evita XL ventilators (Dräger, Germany) in continuous positive airway pressure-assisted spontaneous breathing (CPAP-ASB), biphasic positive airway pressure-assisted spontaneous breathing (BIPAP-ASB) or mandatory minute volume ventilation (MMV) modes. When present, active humidification was turned off at least 30 min prior to measurements.

All aspects of patient care were ultimately decided by the attending physician. To avoid excessive changes in metabolic rate, staff in charge of patients included in the study were encouraged to keep feeding rates, vasopressor infusions and sedation constant 1 h prior to and during measurements. Changes in ventilator settings, endotracheal suctioning, disconnections in the ventilator circuit or mobilization were avoided unless deemed urgently required by medical staff. In case of such events the ongoing measurement was discontinued and restarted after a 15-min resting period. If unexpected deterioration in a patient’s condition required more extensive interventions by clinical staff the measurement was aborted. Multiple measurement series in single patients were performed with a minimum interval of 24 h.

**Fig. 1:**
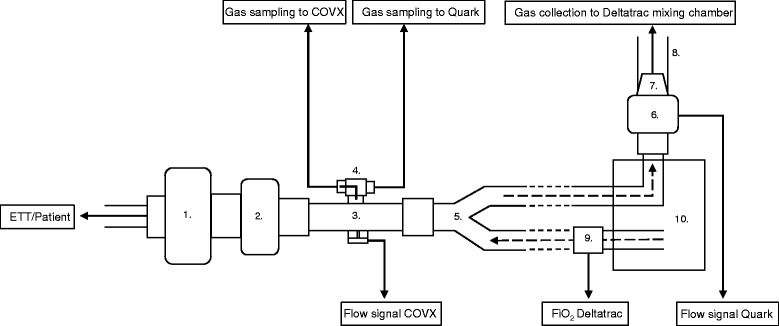
Schematic illustration of instrument connections to the ventilator circuit. 1. HME Filter. 2. Mainstream capnography to ventilator. 3. COVX flowmeter. 4. Three-way stopcock. 5. Y-piece. 6. Quark turbine flowmeter. 7. Adapter from calibration syringe. 8. Corrugated tube. 9. Adapter for Deltatrac FiO_2_ sampling. 10. Evita XL ventilator. *ETT* Endotracheal tube, *FiO*_*2*_ fraction of inspired oxygen

In order to minimize the influence of potential baseline drift in resting energy expenditure (REE), measurements with the study devices and reference method were conducted simultaneously as previously described by Graf et al. [12]. Daily calibrations were performed as recommended by manufacturers or technical manuals. All instruments were connected in parallel to the ventilator circuit. As this required a temporary disconnection of the ventilator and entrainment of room air into the tubing, a 15-min resting period was mandated before commencing measurements. This allowed sufficient time for the mixing chamber of the Deltatrac to equilibrate with alveolar gas from the patient. The flowmeters of the COVX and Quark were connected to the y-piece and expiratory port of the ventilator respectively. The collection tube for expiratory gas to the Deltatrac mixing chamber was then attached to the Quark turbine flowmeter with a plastic adapter. This connection was reinforced with duct tape to prevent gas leaks. Gas sampling lines for both study instruments were connected to the COVX flowmeter using a three-way stopcock, facilitating a switch between devices without disconnecting the ventilator. This setup was approved by the manufacturers of both study devices. An illustration of all connections can be seen in Fig. 1.

Simultaneous measurements with Deltatrac II and either Quark RMR or E-sCOVX were then undertaken for 20 min. After measuring with the first device, another simultaneous measurement was immediately performed with the second device and Deltatrac II. Simple computer randomization was used to determine the order of measurement.

Raw data for all measured parameters were extracted by software applications from each instrument at the highest possible sampling rate. As all three systems provide a different amount of data points over time and treat artifact suppression differently, we used a standardized approach that was communicated to the manufacturers involved prior to the study. When a patient inhales or exhales sharply (as in the case of coughing) gas concentrations and spirometry cannot be properly synchronized, resulting in very low VO_2_/VCO_2_ values. Even though these values are not representative of the patient’s metabolic state, the contribution of single artifact data points potentially offsets the average value of the measurement. The user interface of the E-sCOVX omits these values when displaying the average over time, but when raw data is extracted using the manufacturer’s software these data points are given a value of zero. The Quark attempts to calculate VO_2_/VCO_2_ from available parameters, resulting in artifacts with variable low values. To solve this issue, all data points with the value zero were omitted in calculations of averages from the E-sCOVX module. For the Quark RMR all values which did not fulfil a set of pre-specified criteria (RR >3/<60, tidal volume >0.2/<3 L, fractional content of expired CO_2_ > 0.5/<8 %) were excluded as artifacts. As the Deltatrac is not affected by small variations in breathing patterns, no method of artifact suppression was used.

Using the processed data, average values of VO_2_, VCO_2_, MV and respiratory quotient (RQ) were obtained. REE was calculated using the modified Weir equation, not accounting for nitrogen excretion. Measurements where the reference method registered a mean RQ of <0.6 or >1.2 were discarded.

### Materials

#### Deltatrac II Metabolic Monitor

The Deltatrac Metabolic Monitor uses a mixing chamber technology where all exhaled gas from the patient is collected from the expiratory port of the ventilator. It does not measure flow directly. Gas from the mixing chamber is diluted with room air at a constant flow rate and VCO_2_ is calculated as the product of flow and the concentration of CO_2_ post-dilution (FCO_2_). VO_2_ is then calculated using the Haldane transformation, which assumes that N_2_ is biologically inert and present in the same concentrations in inhaled and exhaled gas. As the denominator in this equation is (1 – FiO_2_), VO_2_ measurements will become increasingly inaccurate as FiO_2_ approaches 1. It is therefore recommended that measurements are restricted to patients with a maximum FiO_2_ of 0.6. It uses a paramagnetic O_2_ analyzer and an infrared CO_2_ analyzer.

### Breath-by-breath instruments

The common feature of breath-by-breath systems is that both flow and gas concentrations are measured over the respiratory cycle. As there is a delay in gas concentration measurements due to the transport time in the sampling lines, flow and gas concentration curves need to be synchronized by software algorithms. VO_2_ and VCO_2_ are then calculated as the product of volumes and concentrations. Due to the difficulties of accurately measuring the small differences in inspired and expired volumes, the assumptions of the Haldane transformation are used to allow for unidirectional flow measurement. This imposes the same limitations on accuracy at higher FiO_2_ levels as with the Deltatrac. As synchronization of gas and flow measurements become increasingly difficult at very high respiratory rates, a maximum RR of 35 was allowed for the purposes of this study.

### Quark RMR

The Quark RMR is a breath-by-breath system for gas exchange measurements. It measures CO_2_ and O_2_ concentrations over the respiratory cycle through a sampling line connected close to the endotracheal tube. Flow is measured using a turbine flowmeter attached to the expiratory port of the ventilator. There is a software application to compensate for bias flow in the ventilator circuit, which has to be set by the user before the start of every measurement. It uses a paramagnetic O_2_ analyzer and an infrared CO_2_ analyzer.

### E-sCOVX

The E-sCOVX module also measures gas exchange breath-by-breath. Gas samples are drawn from a sampling line connected to the flowmeter and analyzed by paramagnetic and infrared methods for O_2_ and CO_2_, respectively. Flow rates are measured by a pneumotach flowmeter connected directly to the Y-piece of the ventilator circuit.

### Statistical analysis

Limits of agreement and bias between VO_2_/VCO_2_ as measured by study instruments (E-sCOVX/Quark RMR) and the reference method (Deltatrac II) were compared using Bland-Altman plots [14]. Agreement of REE values as measured by study devices and the reference method were also analyzed with the Bland-Altman method, although it is a dependent variable calculated from VO_2_ and VCO_2_. A sample size of 50 measurements in at least 20 patients was considered sufficient to determine limits of agreement within ±2.0 standard deviations (SD). Continuous variables with parametric distribution were analyzed for statistical significance using a two-tailed Student’s *t*-test for paired samples and Mann-Whitney *U*-test for non-parametric data, and an α level of ≤0.05 was considered as statistically significant.

## Results

This study was conducted between 27 February 2015 and 5 May 2015. Thirty mechanically ventilated patients were screened for inclusion. Of these, four did not meet the clinical inclusion criteria and three did not give consent. A total of 56 measurements were performed during the study period. Eight measurements were discarded due to a RQ <0.6 (n = 3), gas leaks >10 % of MV (n = 2), RR >35 (n = 1), corruption of saved data (n = 1) and protocol violation (patient was measured twice in <24 h). Forty-eight measurements in 22 patients were included for analysis. Although recruitment fell two cases short of the initial statistical analysis plan, this had no effect on the calculations of limits of agreement or confidence intervals. Patient characteristics are presented in Table 1.

**Table 1:** Patient characteristics

Characteristic	N^a^
Sex (M/F)	17/5
Age	58.6 ± 15.5
BMI	27.6 ± 7.7
Diagnosis	
Septic shock	6
Severe sepsis	2
Respiratory failure	4
Pneumonia	1
Cardiogenic shock	1
Cardiac arrest	2
Other	6
Ventilation mode during measurement	
CPAP-ASB	40
BIPAP-ASB	7
MMV	1
SAPS II^b^	68.4 ± 13.8
SOFA (day of measurement)	10.8 ± 4.3

*BIPAP-ASB* biphasic positive airway pressure-assisted spontaneous breathing, *BMI* body mass index, *CPAP-ASB* continuous positive airway pressure-assisted spontaneous breathing, *MMV* mandatory minute volume ventilation, SAPS Simplified Acute Physiology Score, *SOFA* Sequential Organ Failure Assessment

^a^ Values indicated with ± are means ± standard deviation

^b^ N = 19

**Fig. 2:**
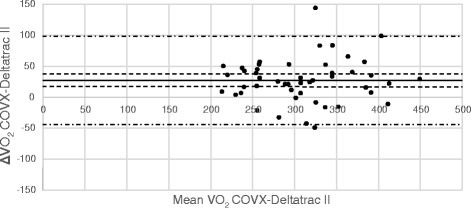
Bland-Altman diagram of VO_2_: E-sCOVX–Deltatrac II. *Solid line*: bias; *dashed lines*: 95 % confidence interval of bias; *semi-dashed lines*: limits of agreement (bias ±2 SD). *VO*_*2*_ oxygen consumption

**Fig. 3:**
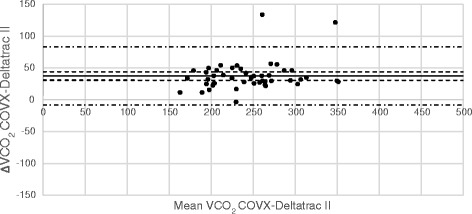
Bland-Altman diagram of VCO_2_: E-sCOVX–Deltatrac II. *Solid line*: bias; *dashed lines*: 95 % confidence interval of bias; *semi-dashed lines*: limits of agreement (bias ±2 SD). *VCO*_*2*_ carbon dioxide production

**Fig. 4:**
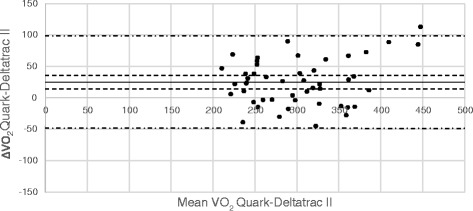
Bland-Altman diagram of VO_2_: Quark RMR–Deltatrac II. *Solid line*: bias; *dashed lines*: 95 % confidence interval of bias; *semi-dashed lines*: limits of agreement (bias ±2 SD). *VO*_*2*_ oxygen consumption

**Fig. 5:**
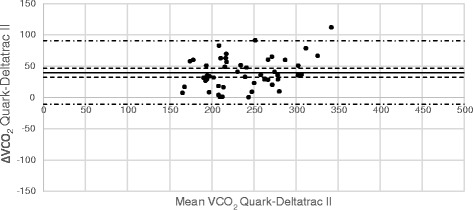
Bland-Altman diagram of VCO_2_: Quark RMR–Deltatrac II. *Solid line*: bias; *dashed lines*: 95 % confidence interval of bias; *semi-dashed lines*: limits of agreement (bias ±2 SD). *VCO*_*2*_ carbon dioxide production

Comparisons between VO_2_ and VCO_2_ measurements from Deltatrac and E-sCOVX/Quark are illustrated in Bland-Altman diagrams (Figs. 2, 3, 4 and 5). Numerical comparisons between instruments and predictive equations are presented in Table 2. There was a significant bias towards higher VO_2_ and VCO_2_ values with both E-sCOVX and Quark RMR as compared to Deltatrac. The precision of measurements with the study instruments and predictive equations expressed as limits of agreement (±2 SD) between methods for VO_2_, VCO_2_ and REE are presented in Table 2. The percentage error (PE = 2 SD/mean value of both methods) for VO_2_ was ±23 % for E-sCOVX and ±25 % for Quark. For VCO_2_, PE was 19 % for E-sCOVX and 21 % for Quark. When comparing REE as measured by Deltatrac II to predictive equations (20 kcal/kg adjusted body weight/24 h and Harris-Benedict equation), PE was ±36 % for 20 kcal/kg and ±29 % for Harris-Benedict.

**Table 2:** Precision and bias of VO_2_ (ml/min), VCO_2_ (ml/min) and REE (kcal/24 h) between methods

Comparison	Mean	Bias	Precision (bias ±2 SD)	PE (±2 SD/mean)
VO_2_: E-sCOVX–Deltatrac	310	+27	–44 to +98	±23
VO_2_: Quark RMR–Deltatrac	301	+25	–49 to +98	±24
VCO_2_: E-sCOVX–Deltatrac	245	+37	–8 to +83	±19
VCO_2_: Quark RMR–Deltatrac	238	+40	–11 to +90	±21
REE: E-sCOVX–Deltatrac	2148	+215	–246 to +675	±21
REE: Quark RMR–Deltatrac	2088	+205	–260 to +670	±22
REE: 20 kcal/kg/24 h Deltatrac	1754	–559	–1199 to 81	±36
REE: HBE* Deltatrac	1773	–371	–926 to +183	±31

* Revised Harris-Benedict equation. *N* = 46

*PE* percentage error, *REE* resting energy expenditure, *SD* standard deviation, *VCO*_*2*_ carbon dioxide production, *VO*_*2*_ oxygen consumption

## Discussion

The purpose of this study was to validate two new instruments for indirect calorimetry against a reference method (Deltatrac II) in critically ill mechanically ventilated patients. Our results showed a systematic bias towards higher VO_2_/VCO_2_ measurements by the breath-by-breath instruments when compared to the Deltatrac II. In our patient population this difference corresponded to approximately 10 % of EE. Both devices displayed a similar variability of individual measurements, corresponding to a percentage error in EE of approximately 20 % compared to Deltatrac.

These results stand in contrast to a previous study by our group which showed no significant difference in mean REE between Quark and Deltatrac [11]. We believe this study is a more accurate representation of the instrument’s properties for three reasons: 1) Measurements with the reference device were performed simultaneously, excluding the possibility that variations in the patient’s metabolic state affects the two measurements differently; 2) The Quark bias flow compensation was more accurate. The level of bias flow was hard to determine prior to the study due to a variable appearance of the flow curve at the expiratory valve. A method of visual assessment for setting the correct compensation was chosen in dialogue with the manufacturer. After all measurements were completed we were informed that our ventilators have no bias flow, and the compensation was retroactively set to zero; and 3) Our previous study did not account for artifacts, which probably offset measured VO_2_/VCO_2_ towards lower values.

It can be questioned whether Deltatrac II is considered a gold standard for gas exchange measurements in this patient group because of its superior accuracy or merely for historical reasons. A compelling argument in support of the mixing chamber method is that it does not require spirometry, only the complete collection of all expired gas. This eliminates a potential source of error in the synchronization of flow and gas concentrations. However, the Deltatrac is an aging piece of equipment and valid questions can be raised concerning the effects of time on its measurement properties. Our own Deltatrac was fully calibrated immediately prior to use and did not show any signs of deterioration.

We can only hypothesize over potential reasons for the systematic bias towards higher REE values with the breath-by-breath instruments. It is unlikely that the effects of humidity or gas leaks contribute to this result as these conditions were tightly controlled during measurements. Minute volumes as measured by the test devices and calculated by Deltatrac were similar, excluding the potential of a systematic overestimation in spirometry. A potential explanation is that the absence of bias flow could interfere with synchronization of gas sampling and flow measurements. At the end of expiration, the y-piece will contain a small volume of alveolar gas. In the presence of bias flow this volume is washed out before the start of inspiration. If it is absent, the first portion of sampled gas during inspiration will be alveolar in composition. As the synchronization algorithm of E-sCOVX assumes a certain inspiratory dead space volume based on wash-out from bias flow, different inputs could result in an overestimation of VO_2_/VCO_2_. It is possible that the two outliers in the E-sCOVX measurements were particularly affected by synchronization issues due to a prolonged period of zero flow at the end of expiration in relation to respiratory rate [15]. No single ventilatory parameter could otherwise be correlated to the degree of overshoot by breath-by-breath instruments. Although the validity of this explanation cannot be tested within the context of this study, it is the most plausible theory available to explain our results. This hypothesis needs to be tested in future validation studies.

Due to the fundamental differences in the technique of measuring gas exchange with a mixing chamber and breath-by-breath instruments, a certain variability in VO_2_/VCO_2_ between simultaneous measurements from these devices is to be expected. This issue of precision is likely dependent on patient–ventilator related factors, such as breathing pattern, respiratory rate and level of sedation. There is currently no consensus regarding the magnitude of bias and limits of agreement which can be considered acceptable in a new device for indirect calorimetry. Black and colleagues propose using a maximum acceptable PE of ±30 % as recommended by Critchley and Critchley [13, 16]. This cutoff is derived from cardiac output monitors, where the gold standard of pulmonary artery catheter thermodilution was assumed to have a precision (coefficient of variation (CV)) of 20 %. The PE derived from a Bland-Altman plot can be regarded as the vector of the precision (CV) of the individual instruments. For a new instrument to have an equal or higher precision than the reference method, the combined precision as calculated from Pythagoras’ theorem cannot exceed 28.3 %. Cecconi et al. have criticized using this cut-off as it is possible for the reference method to have a lower CV than ±20 %, which would result in a smaller acceptable PE [17]. They recommend determining the precision of the reference technique within the context of a given study and calculating the precision of the new technique from the known CV and PE using a Pythagorean approach. We have refrained from using this method as the reference device and the new instruments in our study calculate VO_2_/VCO_2_ from different sets of operands. It is therefore possible that a discrepancy between two methods during an individual measurement reflects a constant bias present under certain conditions rather than an imprecision of the new device.

In a similar study to our own, Graf and colleagues set the recommended limits of agreement at less than ±300 kcals/24 h [18]. This was based on observational data derived from a study of supplemental parenteral nutrition, which related an increased rate of infections to a mean energy deficit of 2300 kcal over 8 days [19]. Assuming a mean REE of 1500 kcal/24 h, this would necessitate a PE <20 %. So far, no breath-by-breath instrument has approached this level of accuracy when compared to a mixing chamber. The goal for manufacturers should ultimately be to achieve the highest attainable agreement between mixing chamber and breath-by-breath instruments. However, the possibility remains that a clinically significantly lower degree of variability than what has so far been demonstrated is unachievable in a modern ICU population that fulfil inclusion criteria similar to those in our study. Narrowing the inclusion criteria could possibly result in better agreement but would limit external validity. Given the greater influence of respiratory patterns on breath-by-breath systems, it is important that clinicians pay close attention to patient–ventilator related factors that may influence measurement results.

The main strength of our study lies in its robust methodological considerations. The technical setup and conditions for simultaneous measurements were rigorously controlled to avoid introducing errors that could have disproportionate effects on certain instruments. Data was collected at the highest possible sampling frequency and adjusted for artifacts. Complete recordings of measurements also enabled the manufacturers of tested instruments to provide feedback from information that cannot be accessed through the standard user interface. In contrast to a mixing chamber, the intricacies of VO_2_/VCO_2_ measurements with breath-by-breath technologies are beyond the scope of clinicians at the bedside. It is therefore crucial that validation studies of new instruments pay close attention to the technical aspects of measurement and potential effects of patient–ventilator interactions.

The limitation of the study lies in the pragmatic approach using the standard ventilator without an intrinsic flow. Therefore, the external validity is limited to the set-up and protocol of the present study. Nevertheless, we believe that our study is the most rigorous evaluation of next-generation indirect calorimeters to date. We recommend that a similar methodology is used in further validation studies. Also, our results highlight the importance of new instruments being thoroughly evaluated in their intended setting before they are used in clinical practice or research.

## Conclusions

Both the E-sCOVX and Quark RMR overestimate VO_2_ and VCO_2_ when compared to Deltatrac II. It is likely that both systematic overestimation and variability in measurements results from patient- and ventilator-related factors that do not affect the mixing chamber technology to a similar extent. Careful consideration to such factors is essential when designing further validation studies of indirect calorimeters. The positive bias corresponding to an overestimation of 10 % in EE is probably not a clinical problem. The variability (PE) of 20 % is more problematic. However, it compares well with variability of measurements in circulatory physiology and it is substantially lower that what is reported for predicted equations.

## Key messages

- In the context of our study, the indirect calorimeters E-sCOVX and Quark RMR measure VO_2_ and VCO_2_ with a bias towards higher values and a variability (percentage error) of approximately ±20 % as compared to Deltatrac II. This is comparable to results commonly accepted in circulatory monitoring.
- The result is probably due to patient- and ventilator-related factors affecting the synchronization of gas and spirometry waveforms during breath-by-breath measurements.
- We propose that a similar methodology is used in future validation studies, and that considerations are taken to the properties of the ventilator used.
- Validating new indirect calorimeters in the ICU setting is essential before they are brought into clinical use.

## Abbreviations

CRRT: Continuous renal replacement therapy
CV: Coefficient of variation
EE: Energy expenditure
FiO_2_: Fraction of inspired oxygen
IC: Indirect calorimetry
ICU: Intensive care unit
MV: Minute volume
PE: Percentage error
REE: Resting energy expenditure
RQ: Respiratory quotient
RR: Respiratory rate
SD: Standard deviation
VCO_2_: Carbon dioxide production
VO_2_: Oxygen consumption
