# Erratum to: ‘Measuring energy expenditure in the intensive care unit: a comparison of indirect calorimetry by E-sCOVX and Quark RMR with Deltatrac II in mechanically ventilated critically ill patients’

**DOI:** 10.1186/s13054-016-1289-2

**Published:** 2016-04-13

**Authors:** Martin Sundström Rehal, Erik Fiskaare, Inga Tjäder, Åke Norberg, Olav Rooyackers, Jan Wernerman

**Affiliations:** Department of Anesthesiology and Intensive Care Medicine, K32, Karolinska University Hospital Huddinge, Hälsovägen 13, 14186 Stockholm, Sweden; Division of Anesthesia and Intensive Care, Department of Clinical Sciences, Intervention and Technology (CLINTEC), Karolinska Institutet, Hälsovägen 13, 14186 Stockholm, Sweden

Unfortunately, the original version of this article [1] contained an error. The corresponding author’s family name and given name were not included correctly and were inverted with given name being ‘Martin Sundström’ and family name being ‘Rehal’. The names are included correctly here, where ‘Martin’ is the given name and ‘Sundström Rehal’ is the family name. This will be corrected in the original article and the copyright line also.
